# Mobius Syndrome and Poland Syndrome Presenting Together in a Single Patient

**Published:** 2015-02-19

**Authors:** Mustafa Chopan, Lohrasb Sayadi, Donald Laub

**Affiliations:** University of Vermont College of Medicine, Burlington

**Keywords:** Mobius syndrome, Poland syndrome, symbrachydactyly, congenital facial nerve paralysis, unilateral pectoralis muscle abnormality

## DESCRIPTION

**Figure 1:**
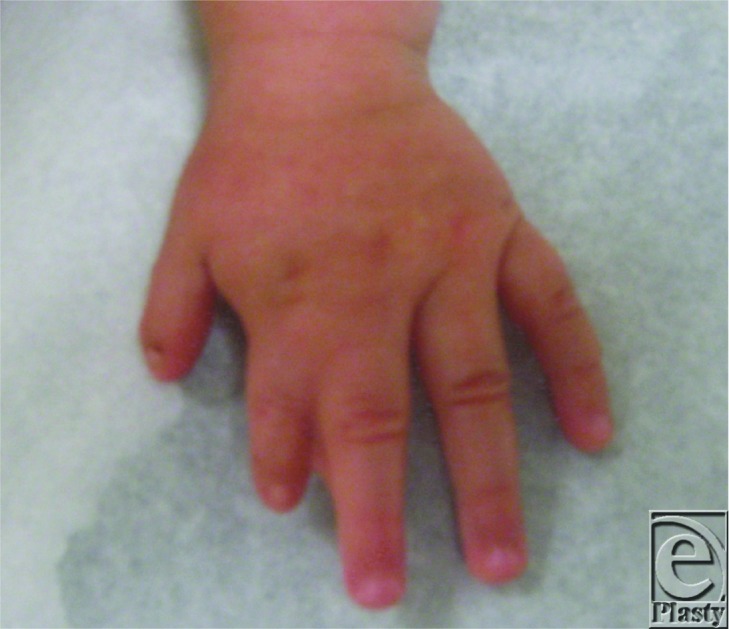
The patient's hand, demonstrating short, webbed fingers.

The patient presented at 16 months of age to the craniofacial clinic with facial nerve abnormalities; a diagnosis of Mobius syndrome was made. At the time of her presentation, she was noted to have short-webbed left index and middle fingers (Fig 1) and an underdeveloped left pectoralis major muscle.

## QUESTIONS

1. **What are Mobius and Poland syndromes?**
2. **What are the suspected causes of these congenital and developmental abnormalities?**
3. **What are the classification systems for Mobius and Poland syndromes?**
4. **What are the treatments for core syndromic defects?**

## DISCUSSION

Mobius (also called Möbius, or Moebius) syndrome refers to a broad spectrum of clinical features associated with congenital facial nerve paralysis. Möbius, in 1892, grouped the sixth and seventh cranial nerve as a single entity. However, the clinical phenotype has since expanded, ranging from bilateral facial and abducens palsy to unilateral facial paralysis. Other multiple cranial nerves (extraocular nerves, glossopharyngeal, vagus, hypoglossal) may also be affected. Mobius syndrome has no gender predilection. Poland syndrome presents with chest wall aplasia and ipsilateral upper extremity anomalies. Poland first described in 1841 a patient with unilateral absence of pectoralis major muscle and symbrachydactyly of the ipsilateral hand. This syndrome has since incorporated other associated anomalies such as hypoplasia of the forearm or breast, rib cage deformities, bilateral epicanthus and talipes equinovarus, and agenesis of the nipple.^1^ Poland syndrome tends to affect more male patients than female patients and the right side more than the left. Stark and Sugarman (1973) first noted the combination of Poland and Mobius syndromes in the same patient.^2^ The estimated prevalence of Mobius-Poland syndrome is 1:500,000, and while some researchers believe the combination to be independent of one another, others believe them to be variations of a similar cause.^1^

The pathogenesis of Mobius syndrome has yet to be determined; however, the triad of genetics, teratogens, and fetal vascular disruptions has been implicated.^3^ While it seems plausible that variations in the timing, location and extent of the vascular insult during embryonic and fetal development give rise to a spectrum of birth defects—the subclavian artery sequence, in particular, has been hypothesized as a unifying theory for Mobius, Poland, Klippel-Feil variants; evidence suggests that Poland syndrome is neither heritable nor the cause of events during pregnancy.^4^^,^^5^ A study of identical twins, in which one was affected with Poland syndrome, leads the authors to propose that Poland syndrome is sporadic and perhaps less affected by genes and teratogens than previously thought.

Facial paralysis in Mobius syndrome may be classified into 4 categories^6^: The first group has an aplasia or hypoplasia of the cranial nerve nuclei. The second, a dysfunction in the peripheral nerve. Third group, a focal necrosis in the region of the brainstem nuclei, and the fourth group, a primary myopathy. Poland syndrome can otherwise be classified by the upper extremity anomalies or the chest wall defects. Historically, the classification was focused on the clinical presentation, depending on the specialty of the treating physician, but now includes degrees of musculoskeletal impairment.

Treatment for facial paralysis in patients with Mobius syndrome is multistaged and may require a variety of surgical procedures, such as fat or muscle transfers, fascia slings, lid weights, and Botox. But the goals of therapy remain to restore facial symmetry, protect the eye and blinking capability, provide oral continence, and allow for the expression of emotion.^3^ Likewise, the repair of chest wall defects in Poland syndrome aims to rectify cosmetic and functional capacity, depending upon the anatomical type and gender.^7^ Single-stage reconstruction is recommended to reduce cost and morbidity, in which the principal surgical goal being chest wall reconstruction with possible augmentation mammoplasty.

Mobius syndrome and Poland syndrome are rare congenital condition affecting the muscles of the face and chest wall, respectively. They have an association, possibly from a common etiology; surgical treatment depends on clinical presentation.
